# Effects of hue and orientation on naturalness and preference: evidence from concrete and abstract art

**DOI:** 10.3389/fpsyg.2026.1809122

**Published:** 2026-06-10

**Authors:** Xiao Wang, Yannan Zhang

**Affiliations:** College of Arts, Northeastern University, Shenyang, China

**Keywords:** abstract art, concrete art, hue, naturalness, orientation, preference

## Abstract

**Introduction:**

In previous research, we found that visual features such as hue and orientation can modulate individuals’ aesthetic preferences. Concrete art contains real world referential representations, whereas abstract art lacks specific referential information. However, it remains unclear whether this modulatory effect differs between these two categories of art.

**Methods:**

The present study employed a three-factor repeated-measures design, manipulating painting category (concrete vs. abstract), hue (original vs. rotated), and orientation (upright vs. inverted), to examine the effects of hue and orientation on perceived naturalness and aesthetic preference across different image categories(concrete art vs. abstract art).

**Results:**

The results showed that: (1) the effect of hue on naturalness and aesthetic preference ratings was stronger for concrete images than for abstract images; (2) the effect of orientation on naturalness and aesthetic preference ratings was stronger for concrete images than for abstract images; (3) perceived naturalness might indirectly moderate the effect on aesthetic preference.

**Discussion:**

Overall, the present study found that the category of artworks (concrete vs.abstract) determines the presence or absence of real-world referential structures, thereby governing the pathway and strength through which visual features influence aesthetic judgment. At the same time, this study also revealed a functionaldissociation between perceived naturalness and aesthetic preference at the psychological mechanism level.

## Introduction

Different groups hold different views on the judgment of beauty, exhibiting distinct individual differences (Jacobi et al., 2024). Due to each person’s level of cultural education, artistic experience, and expertise, it is difficult to define a unified standard for what constitutes beauty (Starr, 2023). In previous neuropsychological research findings, although individuals differ in their subjective definitions of beauty, when faced with the same stimulus materials, groups still show a certain degree of similarity in perceptual processing patterns, physiological responses, and neural activities (Pizzolante et al., 2024). The general regularities of aesthetic judgment and similar behavioral patterns are key areas of focus in empirical aesthetics research (McCrae, 2024).

In the field of visual art aesthetics, the low-level visual features of paintings, such as the basic elements that constitute color: hue, brightness, saturation (Schloss and Palmer, 2011), and the fundamental elements that form spatial organization: spatial structure, compositional balance, and orientation (Nascimento et al., 2017), have become key objects of investigation in aesthetic judgment. Previous studies have manipulated color, composition, or image orientation to examine their effects on the “naturalness perception” of artworks and aesthetic preferences (Vessel et al., 2012). When the color of an image deviates or the spatial structure is inverted, individuals’ aesthetic evaluations of the artwork often decrease (Taniyama et al., 2025).

Aesthetic preference is not only related to the formal features of artworks but also highly correlated with naturalness perception (Albers et al., 2020). From both evolutionary and experiential perspectives, naturalness perception refers to the observer’s subjective judgment of whether a visual stimulus conforms to natural scenes or the statistical regularities of natural vision (Belke et al., 2010). The higher the similarity in statistical features and structure between natural scenes and visual artworks, the more stable the corresponding aesthetic preference tends to be, and the more significantly positive the correlation (Meinel et al., 2024).

### Hue and orientation

In previous studies, color has been identified as a crucial component of an artwork’s composition. When observers were asked to freely adjust the colors of artworks by unfamiliar artists, they tended to converge on the original color compositions (Nakauchi and Tamura, 2022; Nascimento et al., 2017). Prior research has shown that inversion impairs the processing of both isolated features (Bar et al., 2006) and the configuration of features (Gerlach, 2025). When individuals view artworks that are vertical or inverted, participants often prefer the original orientation somewhat, and researchers have named this the tilt effect (Latto et al., 2000). Studies have rotated image orientation while keeping color composition constant, and results show a higher aesthetic preference for upright images (Johnson et al., 2010). Inverting the orientation of artworks destroys the normal aesthetic perception and artistic balance of the artworks (Tatarkiewicz, 2012). Johnson et al. (2010) reported that, among viewers without formal art training, images presented on a computer screen were consistently preferred in their original (upright) orientation compared to rotated versions.

Moreover, when both color composition and spatial arrangement of artworks are manipulated simultaneously to examine their interactive effects on aesthetic judgment (Nakauchi et al., 2022), participants preferred original colors and original spatial structures. Some studies have also found that even when images are divided into small color patches and then randomly combined, participants still show stronger aesthetic preferences for the original artworks (Nascimento et al., 2021). Likewise, when color patches from multiple images are recomposed into collage-like images, viewers continue to prefer the original color compositions over hue-rotated alternatives (Nakauchi and Tamura, 2022). Keeping spatial structure and mean chromaticity constant, researchers rotated original hues using the mean chromaticity method, finding that participants preferred original colors (Nascimento et al., 2017). Segmenting and spatially scrambling original images (Albers et al., 2020) does not alter this preference for the original color distribution. These findings suggest that color and spatial relationships may be processed through relatively independent channels (Gegenfurtner and Kiper, 2003).

### Naturalness and preference

Although different researchers used different methods, these studies found a general phenomenon that maintaining original colors and original spatial structures yielded the highest aesthetic ratings (Nascimento et al., 2021; Palmer et al., 2013). Researchers have named this the original preference effect (Dubois et al., 1999).

Regarding the mechanisms underlying original preference, previous researchers have proposed different explanations (Leynes and Addante, 2016). The mere exposure effect suggests that individuals give more positive evaluations to stimuli consistent with prior experience (Zajonc, 1968). Prototype theory suggests that the perceptual system gives more beautiful evaluations to stimuli closer to category prototypes (Farkas, 2002; Rosch, 1975). Processing fluency theory suggests that stimuli that can be processed more easily and efficiently are more likely to elicit positive aesthetic responses from individuals (Reber et al., 2004).

The closer the image features of visual art are to those of natural scenes (Graham and Redies, 2010), the higher their aesthetic preference ratings (Montagner et al., 2016). Perceived naturalness of low-dimensional images is positively correlated with preference (Kardan et al., 2015). The harmony of artwork composition and the balance of artworks are considered core elements in aesthetic evaluation of images (Tatarkiewicz, 2012). Stable, orderly, and experience-consistent spatial structures or color compositions are often perceived by individuals as more harmonious, natural, and beautiful (Nakauchi et al., 2022). Deviations from typical organization increase individuals’ cognitive load and reduce their aesthetic evaluation (DeLoache et al., 2000).

Previous evidence suggests that preferences for original hues and upright orientations reflect the visual system’s inherent bias toward the naturalness of scenes and stable structures (Hellström, 2024).

### Aims

Combining the above studies, deviation from typical spatial or color configurations tends to reduce individuals’ ratings of perceived naturalness and aesthetic preference for artworks (Vessel and Rubin, 2010). Few studies have examined the effects of both factors simultaneously on different types of artworks (Taylor et al., 2013). Concrete images contain recognizable objects and explicit semantic cues, which may provide higher processing fluency and elicit more stable ratings of perceived naturalness and aesthetic evaluation (Schiller and Gegenfurtner, 2016). However, abstract images lack explicit referential content and objects, and aesthetic judgment relies more on low-level visual features (Reber et al., 2004), with little effect on perceived naturalness and aesthetic evaluation ratings.

The effects of deviation in composition and color from prototypical patterns on perceived naturalness and aesthetic preference may differ between concrete and abstract images, yet this possibility remains underexplored (Russell and Barrett, 1999).

Second, perceived naturalness is closely related to aesthetic preference (Leynes and Addante, 2016). The statistical properties of visual artworks share important similarities with those of natural scenes (Graham and Redies, 2010). In images with rotated hues, color compositions that are perceived as more natural tend to be preferred (Winkielman et al., 2006). This suggests that aesthetic preference may be driven, at least in part, by perceived naturalness. One of the primary aims of the present study is to test this assumption (Spehar et al., 2015).

Hypotheses:

*H1:* Hue has a stronger effect on naturalness and aesthetic preference ratings for concrete images than for abstract images.

*H2:* Orientation has a stronger effect on naturalness and aesthetic preference ratings for concrete images than for abstract images.

*H3:* Perceived naturalness may indirectly moderates the influence on aesthetic preference

## Method

### Participants

M (Mean) represents the arithmetic average of the sample, whereas SD (Standard Deviation) reflects the dispersion of individual values around the mean. Ten male participants (M = 21.80 years, SD = 2.15) and twenty-six female participants (M = 19.85 years, SD = 1.32) took part in the experiment. All participants were Chinese undergraduate students majoring in art at local universities. All were right-handed and reported no history of neurological or psychiatric disorders, nor any hearing impairments. The study was approved by the Ethics Committee of Northeastern University, and written informed consent was obtained from all participants prior to the experiment.

### Experimental design

#### Design

In the present study, rotated hue refers to a 180° shift in hue; inverted orientation refers to a 180° clockwise rotation of the image (Latto et al., 2000). The study employed a three-factor within-subjects (repeated-measures) design, in which the independent variables Category (concrete vs. abstract), Hue (original vs. rotated), and Orientation (upright vs. inverted) were all within-subjects factors, and the dependent variables were ratings of perceived naturalness and aesthetic preference.

#### Material

The images selected for this study are all sourced from WikiArt.org, a recognized non-profit visual art encyclopedia website. All images selected in the study have clear artist attribution and artwork titles. The studies by Marković (2011) and Leder et al. (2012) involved the distinction between concrete/abstract dimensions and the degree of abstraction of artworks, and Pihko et al. (2011) further argued that even the most realistic academic images contain stylized and conventionalized abstract elements, and that concrete and abstract are more like a continuous gradient (Chatterjee et al., 2010). In this study, concrete images refer to artworks that depict recognizable real-world objects with clear referents; abstract images are primarily composed of formal visual elements such as color, shape, and composition (Bimler et al., 2019).

**Figure 1:**
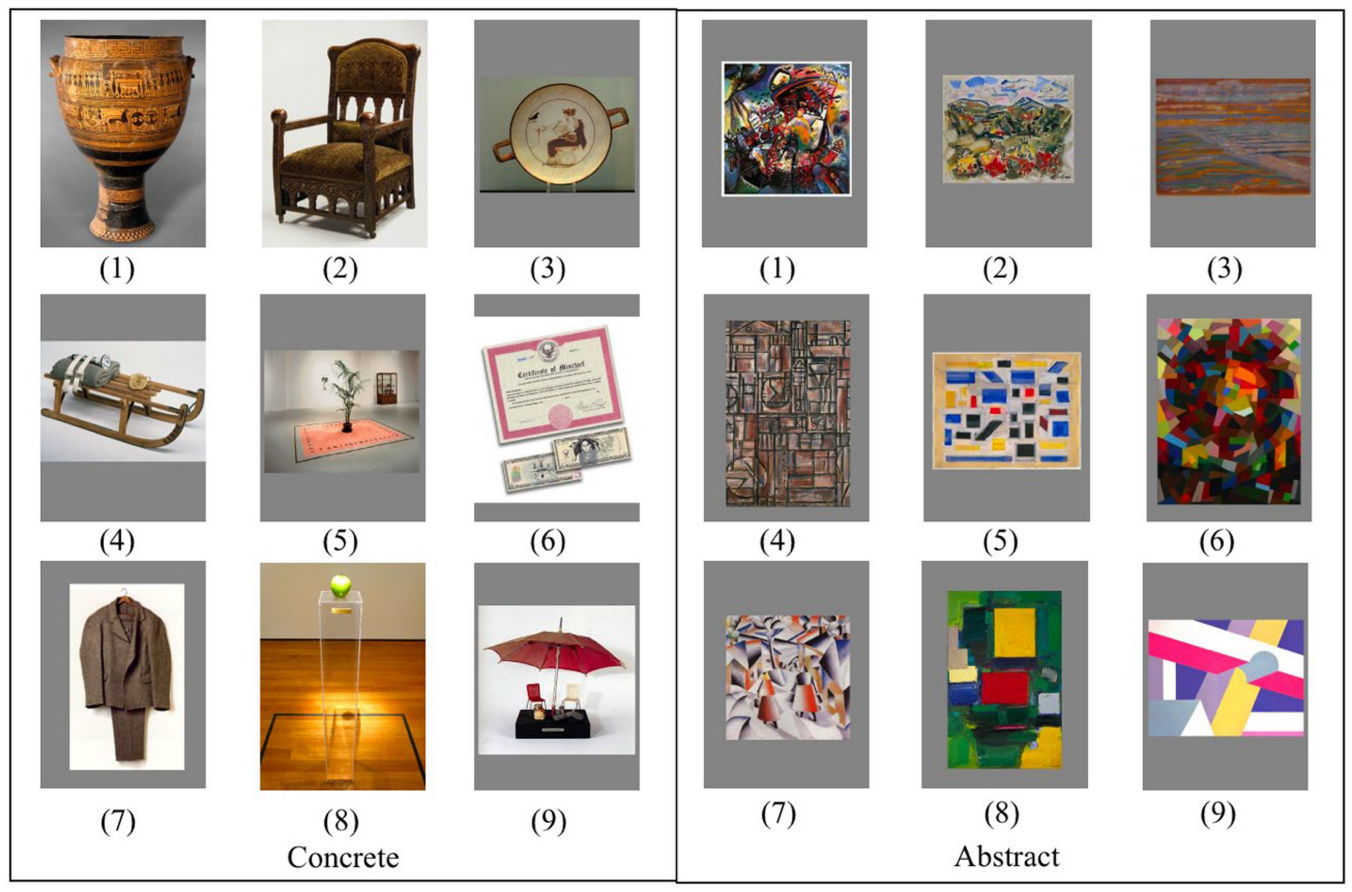
Image sample.

Specifically, the concrete image samples are shown on the left side of Figure 1, consisting of 9 images: (1) Hirschfeld Krater, ancient Greek geometric style, containing funeral rituals and chariot processions; (2) Armchair, a utilitarian artwork; (3) Cylix of Apollo, a red-figure pottery cup depicting the god Apollo; (4) Sled, composed of ready-made objects such as a wooden sled, blanket, flashlight, and fat. (5) Tapis de Sable, a carpet made of sand.(6) Certificate of Mischief Nation, consisting of a certificate and two banknotes. (7) Felt suit, a suit made of felt. (8) Apple, a real apple. (9) Raining Cats and Dogs, cats, dogs, and an umbrella.

The images are derived from Conceptual Art, Fluxus, Happening, Performance Art, Installation Art, Social Sculpture. These contemporary art forms do not primarily use depiction as their means, but a large number of works among them use ready-made objects (e.g., Beuys’ sled, felt suit, Yoko Ono’s apple). These objects themselves are concrete objects in the real world and have clear referents. The abstract images are shown on the right side of Figure 1, consisting of 9 images. (1) Moscow I, non-concrete colors and dynamic forms expression; (2) Tunk Mountains, Autumn, Maine, vibrant brushstrokes and geometric color patches; (3) View from the Dunes with Beach and Piers, horizontal color patches and pointillism; (4) Structure, composed of geometric shapes, lines, and symbols; (5) Untitled, pure colors and geometric forms; (6) Rosace II, primarily composed of abstract color and form; (7) Morning in the Village after Snowstorm, composed of geometric shapes such as cubes and rectangles; (8) The Gate, composed of active color patches and rectangles; (9) Transverse Beams, highly geometricized forms. The images are derived from Abstract Expressionism, Neo-Impressionism, Constructivism, Neoplasticism, De Stijl, Cubism, Cubo-Futurism, Russian Avant-Garde. The works do not imitate the real world; color, line, shape, and composition themselves constitute the aesthetic subject. The distinction between concrete and abstract images reflects differences in concrete structure, and the titles, artists, genre and dates of the image sources are listed in Table 1.

**Table 1:** Source of image sample.

Concrete	Title	Artists	Correct genre	Date
1	Hirschfeld Krater	Ancient Greek Pottery	Greek and Roman Art	c.735 BC
2	Armchair	Louis Comfort Tiffany	Art Nouveau furniture design	1892
3	Cylix of Apollo	Ancient Greek Pottery	Mythological painting	c.470 BC
4	Sled	Joseph Beuys	Conceptual Art, Installation Art	1969
5	Tapis de Sable	Marcel Broodthaers	Conceptual Art, Installation Art	1974
6	Certificate of Mischief Nation	Kent Monkman	Contemporary Indigenous Art, Performance Art, Installation Art	2013
7	Felt suit	Joseph Beuys	Fluxus, Conceptual Art	1970
8	Apple	Yoko Ono	Fluxus, Conceptual Art, Instructional Art (Happening)	1966
9	Raining Cats and Dogs	Robert Filliou	Fluxus, Conceptual Art	1969

Abstract	Title	Artists	Correct genre	Date
1	Moscow I	Wassily Kandinsky	Early Abstract Art, Expressionism	1916
2	Tunk Mountains, Autumn, Maine	John Marin	American Modernism, Watercolor abstraction	1945
3	View from the Dunes with Beach and Piers	Piet Mondrian	Post-Impressionism (transitional phase toward abstraction)	1909
4	Structure	Joaquin Torres García	Neoplasticism, Constructivism, Universal Constructivism	1931
5	Untitled	Bart van der Leck	De Stijl, Neoplasticism, Abstract Art	1917
6	Rosace II	Otto Freundlich	Abstract Art, Expressionism, early Constructivist abstraction	1941
7	Morning in the Village after Snowstorm	Kazimir Malevich	Suprematism	1913
8	The Gate	Hans Hofmann	Abstract Expressionism	1959–1960
9	Transverse Beams	Patrick Henry Bruce	Cubism, Synchromism	1932

**Figure 2:**
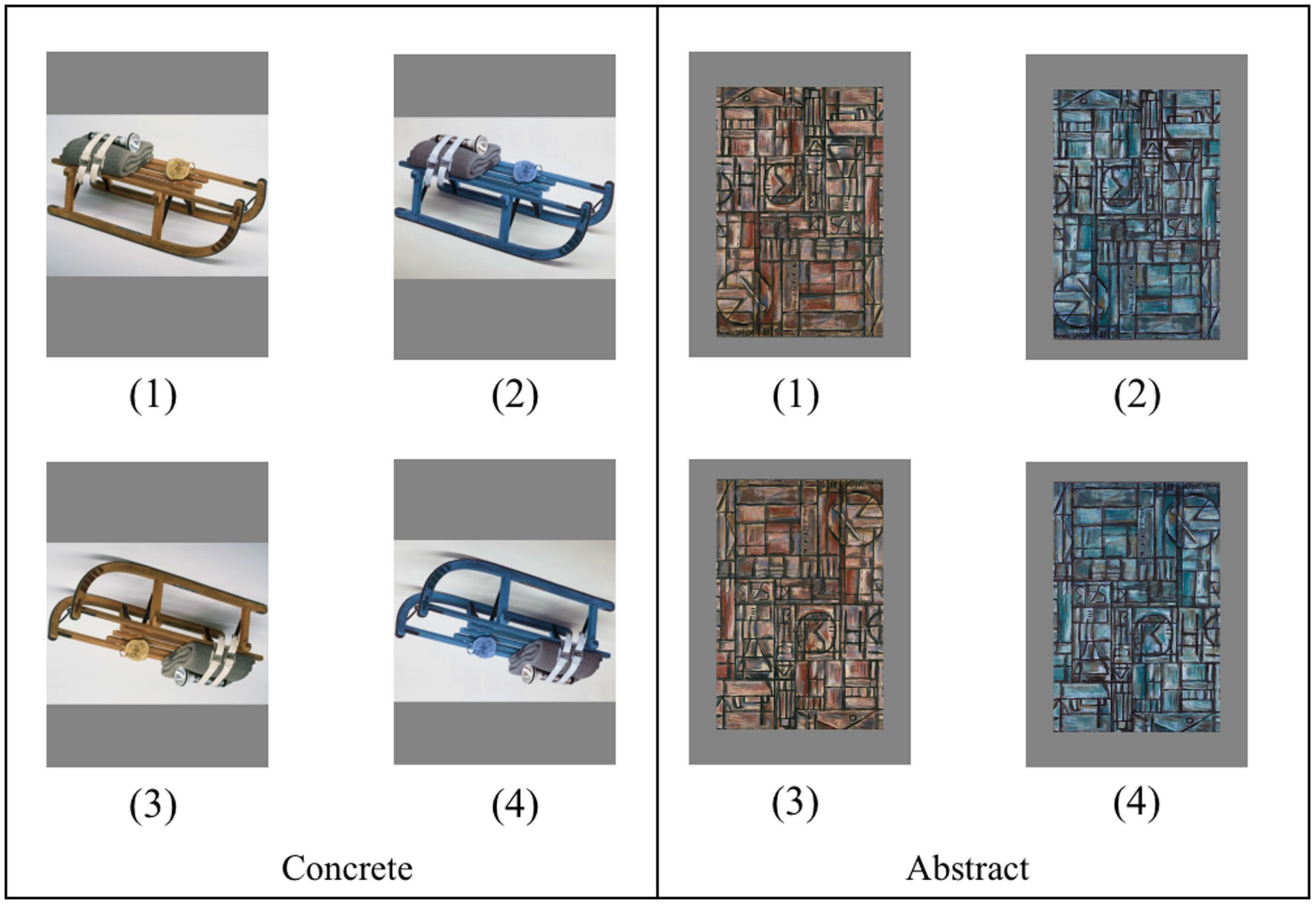
Operation image sample note. (1) Original hue and upright orientation; (2) rotated hue and upright orientation; (3) original hue and inverted orientation; (4) rotated hue and inverted orientation.

As illustrated in Figure 2, four stimulus conditions were created by manipulating hue and orientation: (1) original hue and upright orientation, representing the unaltered image condition; (2) rotated hue and upright orientation, in which the hue of the image was rotated by 180° while the spatial orientation remained upright; (3) original hue and inverted orientation, in which the hue remained unchanged while the image orientation was rotated by 180°; and (4) rotated hue and inverted orientation, in which both hue rotation and orientation inversion were applied simultaneously.

#### Procedure

**Figure 3:**
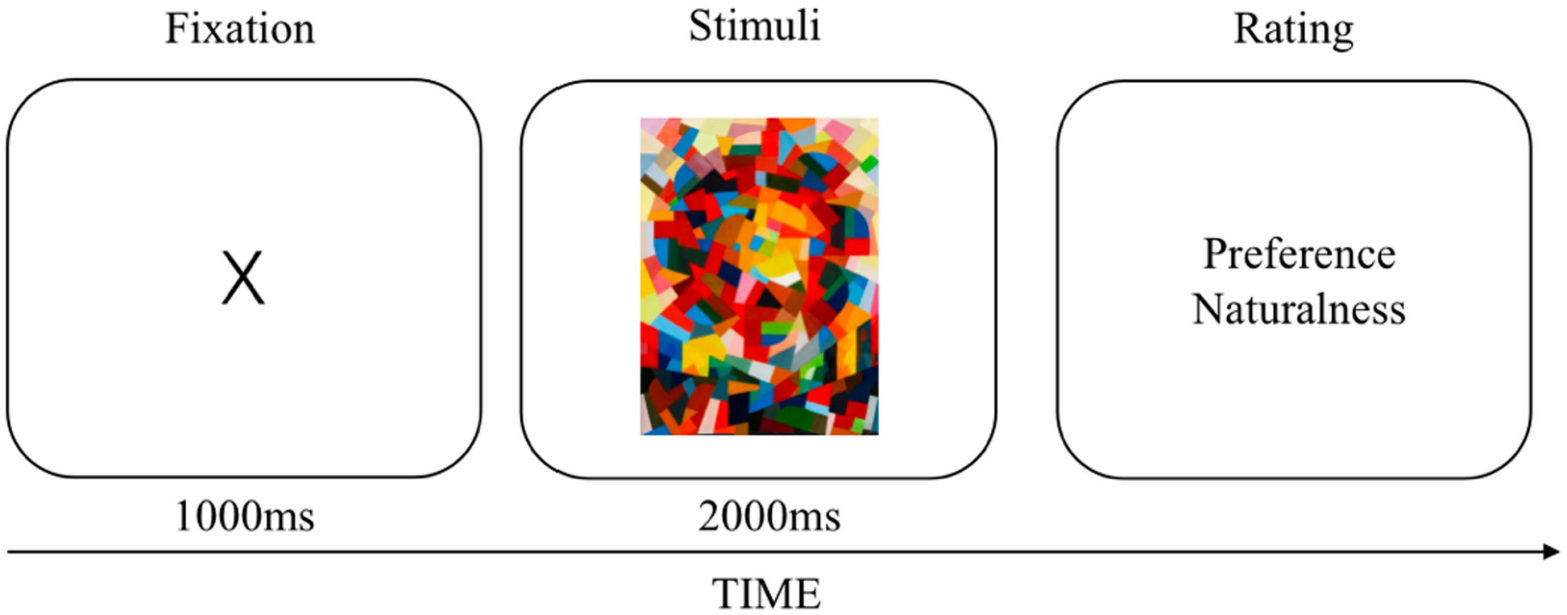
Experimental procedure.

As shown in Figure 3, the entire experiment was conducted in a quiet laboratory equipped with soundproofing facilities. Participants entered the laboratory one at a time, sat down in front of a computer, and rested for 1–2 min to acclimate to the environment. At the same time, the experimenter communicated with the participants to ensure that they were emotionally stable and comfortable, and informed them that they had the right to withdraw from the experiment at any time.

Participants read the written instructions on the screen: (1) They would view a series of images and were asked to rate them based on their subjective feelings; there were no right or wrong answers. (2) “Preference” referred to the participant’s aesthetic liking of the image, with higher ratings indicating greater preference; “Naturalness” referred to the subjective impression that an image looks natural rather than artificial, for example, whether the colors and the orientation of the scene look like what one would see in the real world. (3) They were to use the keyboard number keys 1–5 to rate, with 1 indicating “very unnatural” or “very low preference,” and 5 indicating “very natural” or “very high preference.” (4) Each image was presented for 2 s, but there was no time limit for the ratings; however, they were asked to respond as quickly and spontaneously as possible. To help participants become familiar with the procedure, three fixed practice trials were conducted first, using images that did not appear in the formal experiment. After the practice, the experimenter confirmed that the participants had fully mastered the requirements before proceeding to the formal experiment. Before the formal experiment began, the experimenter told the participants that the experiment involved rating some images based on their own subjective feelings, but did not inform them of the true purpose of the experiment. The entire experimental procedure was programmed using Microsoft PowerPoint, and response data were recorded in an Excel file. Because response times varied across participants, the total duration of the experiment, including the practice trials, ranged from approximately 8 to 10 min per participant.

## Results

### Mean of preference and naturalness

As shown in Table 2, for the three-factor repeated measures of different categories (concrete images vs. abstract images), hue (original hue vs. rotated hue), and orientation (upright vs. inverted), the mean (M) and standard deviation (SD) of participants’ ratings of naturalness and preference for images under each condition. For example, under the condition of original hue and upright orientation for concrete images, the arithmetic mean rating of the 9 concrete images under the original hue and upright orientation condition was 3.725, and the standard deviation was 0.667. The results suggest systematic variations in ratings across these three factors: both hue manipulation and orientation manipulation influenced participants’ evaluations, and under the four conditions of (1) original hue and upright orientation; (2) rotated hue and upright orientation; (3) original hue and inverted orientation; (4) rotated hue and inverted orientation, the pattern was (1) > (2) > (3) > (4).

**Table 2:** Mean of preference and naturalness.

Category	Hue	Orientation	Naturalness		Preference	
			*M*	*SD*	*M*	*SD*
Concrete	Original	Upright	3.725	0.667	3.299	0.536
	Rotated	Upright	3.216	0.679	3.086	0.711
	Original	Inverted	3.003	0.697	2.954	0.564
	Rotated	Inverted	2.617	0.554	2.710	0.578
Abstract	Original	Upright	2.667	0.651	2.654	0.669
	Rotated	Upright	2.728	0.691	2.698	0.723
	Original	Inverted	2.694	0.678	2.611	0.684
	Rotated	Inverted	2.651	0.754	2.716	0.765

### Results of there-way repeated-measures ANOVA for preference rating and naturalness rating

As shown in Table 3, a repeated-measures ANOVA revealed significant main effects of Category (concrete vs. abstract), Hue (original vs. rotated), and Orientation (upright vs. inverted). For naturalness ratings, the main effects of Category, Hue, and Orientation were all significant. The three-way interaction among Category * Hue * Orientation was not significant. Significant two-way interactions were found for Category * Hue and Category * Orientation, whereas the Hue * Orientation interaction was not significant. Further simple effects analyses are therefore required.

**Table 3:** Analysis of variance.

Conditions	Naturalness				Preference			
	*df*	*F*	*p*	*η* ^2^ *p*	*df*	*F*	*p*	*η* ^2^ *p*
Category (concrete/abstract)	1	23.672	<0.001	0.403	1	11.549	0.002	0.248
Hue (ORIGINAL/ROTATED)	1	11.455	0.002	0.247	1	2.937	0.095	0.077
Orientation (Upright/Inverted)	1	37.713	<0.001	0.519	1	18.796	<0.001	0.349
Category*Hue	1	33.693	<0.001	0.490	1	13.335	0.001	0.276
Category*Orientation	1	38.859	<0.001	0.526	1	22.683	<0.001	0.393
Hue*Orientation	1	0.014	0.905	0.000	1	0.075	0.786	0.002
Category*Hue*Orientation	1	2.362	0.133	0.063	1	0.657	0.423	0.018

For preference ratings, the main effects of Category and Orientation were significant, whereas the main effect of Hue was not significant. Two-way interactions for Category * Hue and Category * Orientation was significant, but the Hue * Orientation interaction was not significant, and the three-way interaction among Category * Hue * Orientation was not significant. These results warrant further simple effects analyses.

### Simple effects analysis

**Figure 4:**
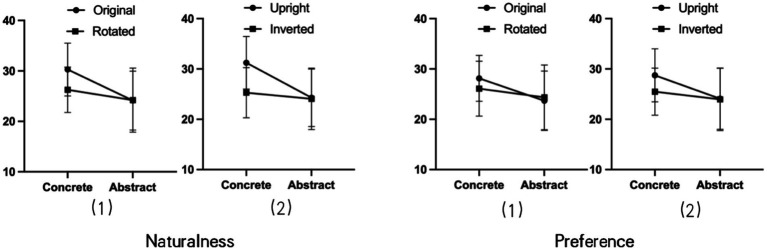
Simple effects analysis.

As shown in Figure 4, the effects of hue and orientation on naturalness ratings both occurred in concrete images: (1) under the concrete image condition, naturalness ratings for original hue were significantly higher than those for rotated hue (mean difference *ΔM* = 4.028, *p* < 0.001), while no significant difference was found for abstract images (*ΔM* = −0.083, *p* = 0.891); (2) under the concrete image condition, naturalness ratings for upright orientation were significantly higher than those for inverted orientation (*ΔM* = 5.944, *p* < 0.001), while no significant difference was found for abstract images (*ΔM* = 0.222, *p* = 0.511).

Similarly, the effects of hue and orientation on preference ratings both occurred in concrete images: (1) under the concrete image condition, preference for original hue was significantly higher than that for rotated hue (*ΔM* = 2.056, *p* = 0.002), while no significant difference was found for abstract images (*ΔM* = −0.667, *p* = 0.169); (2) under the concrete image condition, preference for upright orientation was significantly higher than that for inverted orientation (*ΔM* = 3.250, *p* < 0.001), while no significant difference was found for abstract images (*ΔM* = 0.111, *p* = 0.797).

Overall, there is a consistent and similar effect between naturalness and preference for concrete and abstract images. However, the results do not indicate a direct unidirectional causal relationship between naturalness and aesthetic preference; in real life, individuals’ familiarity with artworks, as well as some typical features of the artworks themselves, or long-term exposure effects, may also explain the obtained findings.

## Discussion

### Hue has a stronger effect on naturalness and aesthetic preference ratings for concrete images than for abstract images

Consistent with Hypothesis 1, the original hue enhanced the perceived naturalness of figurative artworks and also increased the aesthetic ratings of figurative art (Linhares et al., 2008). In abstract images, the same manipulation had less effect on naturalness and preference ratings (Chamberlain et al., 2022; Francuz et al., 2018). In previous studies, the manipulation of hue by varying degrees of rotation yielded highly consistent findings, and prototype theory is often used to explain this phenomenon, both receiving substantial empirical support (Nakauchi et al., 2022). Specifically, art contains recognizable objects and stable spatial relationships, providing a reference to the real world and real-world prototypes (Graham and Redies, 2010). Under the influence of developmental evolution and experiential learning, the human visual system has gradually adapted to the statistical regularities of the natural environment, resulting in higher ratings for such art (Simoncelli and Olshausen, 2001). Hue deviation disrupts perceptual rationality, reduces perceived naturalness, weakens processing fluency, and lowers aesthetic preference (Augustin et al., 2008). Abstract images are not intended to represent the structure of the real world; the formal organization of abstract art lacks stable reference points, making it difficult for observers to perceive natural regularities (Redies, 2008), thereby reducing aesthetic preference evaluations (Chenier and Winkielman, 2018).

### Orientation has a stronger effect on naturalness and aesthetic preference ratings for concrete images than for abstract images

Consistent with Hypothesis 2, studies manipulating orientation using different rotation angles also consistently show that original orientations are preferred over rotated versions (Palmer et al., 2013). People have a mental “canonical perspective” for everyday objects, for which recognition speed is fastest and evaluation is highest (Baddeley and Long, 1981). In studies on the aesthetic evaluation of Mondrian paintings, researchers first reported the “aesthetic oblique effect”: when the constituent lines of a painting are horizontal and vertical, aesthetic preference is higher than for vertical orientations (Latto et al., 2000). Orientation judgment plays a key role in whether an image contains recognizable real-world objects, and the accuracy of orientation judgment is highly correlated with the aesthetic evaluation of the image (Mather, 2012). According to the mere exposure effect, individuals prefer stimuli they are frequently exposed to. In concrete art, people in real life are generally exposed to upright orientations, and the brain processes them with greater fluency; during long-term exposure, processing occurs in immediate and automatic encoding (Bornstein and Craver-Lemley, 2022). Abstract art, however, lacks clear referents and identifiable information from reality, so aesthetic judgments rely more on low-level visual features (Pelowski et al., 2025), making it difficult to form stable mental representations for comparison, and thus the difference is not significant (Ball et al., 2018).

### Perceived naturalness may indirectly moderates the influence on aesthetic preference

Consistent with Hypothesis 3, naturalness ratings and preference ratings showed similar trends. Original hue paintings or original orientation paintings were perceived as more natural and received higher ratings. This is consistent with previous findings that when a painting is less natural, it is less preferred (Fernandez and Wilkins, 2008). Of course, naturalness also has multiple dimensions; it depends on how close something is to nature, or how far it is perceived from the artificial (Vessel et al., 2012). Perceptions of naturalness may also increase with long-term exposure, and the mere exposure effect may increase familiarity independently of the physical properties of the stimulus (Siipi, 2008). That a specific painting is more familiar to the observer may be one reason for its higher naturalness rating (Zajonc, 1968). At the same time, original paintings tend to have higher typicality; they better conform to the viewer’s prototype representation of that genre or theme. High typicality facilitates processing fluency and enhances aesthetic preference (Reber et al., 2004). Typical things tend to be perceived as more natural, which may increase both naturalness ratings and preference ratings (Winkielman et al., 2006). Therefore, perceived naturalness may indirectly moderate the effect on aesthetic preference (Taniyama et al., 2025).

## Conclusion

The results of this study indicate that the human visual system has gradually adapted to the statistical regularities of natural environments over the course of evolution, and that visual features consistent with natural structure are more readily processed and elicit more positive evaluations. Hue and orientation play a key role between perceived naturalness and aesthetic preference. Naturalness is reflected in judgments of the degree of closeness to “nature” or distance from “artificial,” and evaluations of naturalness often precede aesthetic preference and provide a perceptual foundation for it; evaluations of naturalness may moderate the formation of aesthetic preference.

Overall, the present study finds that artwork category (concrete vs. abstract), by determining whether a real-world referential structure is available, governs the pathway and strength through which visual features influence aesthetic judgment. At the same time, it reveals a functional dissociation between perceived naturalness and aesthetic preference at the level of psychological mechanisms.

### Limitation

This study still has several limitations worth further discussion, particularly regarding the selection of experimental stimulus materials and methodological control.

First, although this study aimed to compare the perceived naturalness and aesthetic preferences between concrete and abstract art under manipulations of hue and orientation, the two categories of stimuli were not fully homogenized in terms of visual attributes and medium forms. Concrete art is presented as three-dimensional objects with clear everyday object properties, whereas abstract art mainly consists of two-dimensional abstract paintings. Reducing concrete stimuli to two-dimensional paintings, while aligning them formally with abstract paintings, would deprive the concrete stimuli of key ecological information (such as volume, manipulability, and real-world contextual associations), thereby altering their original attributes as concrete art and reducing construct validity. Conversely, elevating abstract stimuli to three-dimensional objects, such as abstract sculptures or craft objects, may lead participants to perceive the presence of concrete information within them, thereby confounding the experimental variables and resulting in inaccurate categorization.

Second, concrete stimuli tend to have stronger real-world referentiality and semantic familiarity. Participants may form judgments of “natural” or “unnatural” more readily because they can quickly recognize concrete objects. In contrast, abstract works lack clear semantic cues, and the criteria for perceiving naturalness in abstract stimuli may be inherently more ambiguous. The differences in naturalness ratings observed in this study may partly originate from differences in stimulus familiarity and semantic identifiability, rather than solely from variations in visual form features.

In addition, potential differences across stimulus categories in the perception of artistry may also influence aesthetic judgments. When faced with everyday objects, participants may not always consider them as artworks, whereas abstract paintings are more readily categorized as art. Such differences in the perception of artistic status may further affect participants’ judgments of artificiality, naturalness, and aesthetic preference.

Therefore, future research should further strengthen the strict control of stimulus materials. For example, studies could use only a single category of materials, or simplify the experimental content to allow more refined comparisons between concrete and abstract arts. It is also important to match stimuli as closely as possible on confounding factors such as size, complexity, luminance, familiarity, and semantic information. Future studies could further introduce variables such as familiarity, perceived artistry, and semantic identifiability as covariates or mediators, in order to more accurately reveal the psychological mechanisms through which visual features influence naturalness perception and aesthetic preferences.

Despite the above limitations, this study provides preliminary evidence for how visual features affect naturalness perception and aesthetic preferences across different art categories, and offers a reference for future efforts to establish more rigorous and systematic experimental paradigms in the cognitive science of art.

## Data Availability

The original contributions presented in the study are included in the article/Supplementary material, further inquiries can be directed to the corresponding author.
